# Senegenin Attenuates Hepatic Ischemia-Reperfusion Induced Cognitive Dysfunction by Increasing Hippocampal NR2B Expression in Rats

**DOI:** 10.1371/journal.pone.0045575

**Published:** 2012-09-21

**Authors:** Weibin Xie, Yan Yang, Xiaoping Gu, Yaguo Zheng, Yu-e Sun, Ying Liang, Jinhua Bo, Zhengliang Ma

**Affiliations:** 1 Drum Tower Clinical College of Chinese and Western Integrative Medicine, Nanjing University of Chinese Medicine, Nanjing, Jiangsu Province, China; 2 Department of Anesthesiology, Affiliated Drum-Tower Hospital, Medical College of Nanjing University, Nanjing, Jiangsu Province, China; Hôital Robert Debré, France

## Abstract

**Background:**

The root of Polygala tenuifolia, a traditional Chinese medicine, has been used to improve memory and intelligence, while the underlying mechanisms remain largely unknown. In this study, we investigated the protective effects of senegenin, an component of Polygala tenuifolia root extracts, on cognitive dysfunction induced by hepatic ischemia-reperfusion.

**Methodology/Principal Findings:**

Initially, we constructed a rat model of hepatic ischemia-reperfusion (HIR) and found that the memory retention ability of rats in the step-down and Y maze test was impaired after HIR, paralleled by a decrease of N-methyl-D-aspartate (NMDA) receptor NR2B subunit mRNA and protein expressions in hippocampus. Furthermore, we found that administration of senegenin by gavage attenuated HIR-induced cognitive impairment in a dose and time dependent manner, and its mechanisms might partly due to the increasing expression of NR2B in rat hippocampus.

**Conclusions/Significance:**

Cognitive dysfunction induced by HIR is associated with reduction of NR2B expression. Senegenin plays a neuroprotective role in HIR via increasing NR2B expression in rat hippocampus. These findings suggest that senegenin might be a potential agent for prevention and treatment of postoperative cognitive dysfunction (POCD) or other neurodegenerative diseases.

## Introduction

The Polygala tenuifolia root is an oriental herbal medicine, which has been traditionally used to treat patients with insomnia, neurosis, or dementia [1]. Several studies have reported that the Polygala tenuifolia root extracts have neuroprotective and neuroregenerative effects [2], [3]. These extracts can enhance cognitive functions in elderly individuals and promote memory enhancement in healthy adults [4], [5].

Senegenin, which is also called tenuigenin, is an effective component of Polygala tenuifolia root extract. The chemical structure of senegenin is as follows: (2β,3β,4α,12α)-12-(Chloromethyl)-2,3-dihydroxy-27-norolean-13-ene-23,28-dioic acid (molecular formula: C30H45ClO6; molecular mass: 537; lipophilic and hydrosoluble) [6], [7]. Pharmacological data indicated that tenuigenin displayed antiapoptotic and antioxidative activity in hippocampal neurons due to scavenging of intracellular reactive oxygen species, regulating Bcl-2 family and suppressing caspase-3 activity [7]. Some studies also showed that tenuigenin could protect neuronal cells against toxins such as 6-OHDA [8] or H2O2 [9]. In addition, in vitro studies have indicated that tenuigenin treatment suppress secretion of amyloid β protein [10], [11] and attenuate its cytotoxicity [12], which is a pivotal pathological factor in Alzheimer's disease (AD). However, the molecular mechanism underlying these effects remains unknown.

Hepatic vascular occlusion is a frequent procedure during liver surgery to treat patients with end-stage liver disease. The resultant hepatic ischemia-reperfusion (HIR) may result in cell death, which initiates a chain of events that culminate in systemic inflammatory response and multi-organ failure [13]. The brain is one of the most susceptible targets for distant organ damage induced by hepatic ischemia-reperfusion [14]. It has been previously reported that peripheral inflammation can profoundly affect the function of central nervous system, including memory and cognition [15]. Moreover, increasing evidences have indicated that surgical trauma, resulting in the release of inflammatory mediators, is a prominent risk factor for the development of postoperative cognitive dysfunction (POCD) [16], [17]. Nevertheless, the definite mechanism of neuron impairment in POCD remains unclear and there is little information available about how the central nervous system (CNS) recognizes and responds to inflammatory mediators.

In the present study, with a model of hepatic ischemia-reperfusion in rats, we aim to investigate the protective effects of senegenin in hepatic ischemia-reperfusion induced cognitive dysfunction and whether increasing NR2B expression in hippocampus is involved in the phenomenon.

## Materials and Methods

### Animal

Animal experiments were performed following the guidelines in the Guide for the Care and Use of Laboratory Animals published by the US National Institutes of Health (NIH publication No. 85-23, revised 1985) and was approved by the Ethics Review Board for Animal Studies of Nanjing Drum Tower Hospital (DTH ERBA 66.01/028C/2011). Adult male Sprague-Dawley rats weighting 200–250 g were housed four to a cage and acclimatized before experiment under conditions of controlled temperature (23∼25°C) and illumination (12-hour light-dark cycle) and allowed free to standard rat chow and sterile water. After excluding rats abnormal in behavioural memory test, one hundred and twenty rats were randomly divided into five groups: sham, HIR, and HIR+ sen 15, 30 and 60 mg/kg groups (n = 24, respectively). Rats in the HIR+ sen 15, 30 and 60 mg/kg groups were administered senegenin by gavage at dose of 15, 30 and 60 mg/kg once per day started one day before surgery and thereafter continuously until day 7 after operation. Senegenin (purity>98%) was purchased from Nanjing Zelang pharmaceutical science and technology Co., Ltd (Jiangsu province, China). The sham and HIR groups received the same volume of saline.

### Liver ischemia-reperfusion

Rats were fasted for 12 hours but allowed free access to water before the induction of anesthesia. They were anaesthetized with sodium pentobarbital (50 mg/kg body weight, i.p.). Laparotomy was performed through a midline incision. The ligamentous attachments from the liver to the diaphragm were severed, and the liver was exposed. Ischemia of the median and left lateral lobes of the liver was produced by clamping the corresponding vascular pedicle containing the portal vein and hepatic artery branches by using an atraumatic microvascular clamp. This method produced ischemia to the left and median lobes of the liver (about 70% of the liver) while leaving the blood supply to the right and caudate lobes uninterrupted [18]. The vascular clamp was removed and reperfusion was allowed after 60 minutes of ischemia. All procedures were performed using sterile technique.

### Assessment of Learning and Memory

The step down and Y maze (both supplied by Chengdu TME Technology Co., Ltd, Sichuan province, China) tests were carried out in rats before and at day 1, 3, 5 and 7 after operation.

#### Step down test

It's a passive avoidance test to assess the behavioral reaction of rats that includes training process and formal testing. The apparatus consisted of five boxes equipped with copper bars at the bottom and one plastic platform. Each rat was individually placed on the copper bars in every box and 3-min later the copper bars were electrified. Electricity-shocks rats jumped upon the plastic platform to avoid the electric current. The rats then descended again to the copper bar after some minutes, and quickly jumped onto the plastic platform again after being shocked again. All the rats were trained by this process for 5-min. After 24 h, rats were individually placed on the plastic platforms of the boxes while the copper bars were again electrified. The interval between placement on the platform and step-down onto the copper bar was recorded as “Escape latency”, which was used as an index for evaluating the ability of memory retention. Shorter escape latency indicated poor memory ability [19].

#### Y maze

For the procedure, a computer-controlled Y-maze consisting of three equal arms (30×15×15 cm) with a stainless-steel grid floor was used as described earlier [20]. At the beginning, a foot shock (0.7–1.3 mA, depending on individual sensitivity) was given in the “stem” arm and the animal had to escape into the right alley (correct run, no foot shock in this arm), whereas entry into the left alley (error) was punished by further foot shock. Abnormal rats were excluded after 3 trials, and then numbers of error in 5 min before and at day 1, 3, 5, 7 after operation were evaluated.

Rats were decapitated after completion of the memory tasks. Liver tissues were collected from the rats and stored at −20°C for histopathological examination.Hippocampus was quickly removed and immediately cooled in liquid nitrogen and kept at −80°C for messenger RNA (mRNA) and western blot analysis.

### Histopathological examination

Liver tissue samples were stored in 10% formalin solution and routinely processed into 5 µm paraffin sections. Sections for histopathological analysis were stained with hematoxylin and eosin. Images (×100) of liver sections was captured with Olympus microscope equipped with digital camera. Histopathological examination was performed by a pathologist who specialized in this field for observing pathological injury degree.

### RT-PCR analysis

At day 1, 3, 5 and 7 postoperation, NR2B mRNA levels were measured in hippocampus of three rats extracted randomly from each group using reverse transcription-polymerase chain reaction (RT-PCR) analyses.

Total RNA was isolated and purified with an Rneasy Mini Kit. cDNA was produced using PrimeScripttm RT reagent Kit (TaKaRa). A Power STBR Green Master Mix was used for RT-PCR analysis (the ABI Prism 7500 Detection system). Primers for NR2B were 5′-GCATTCCTACGACACCTTCG-3′ (upstream) and 5′-GACCACCACTGGCTTATTGG-3′ (downstream) [21], and primers for GAPDH were 5′-AACGACCCCTTCATTGAC-3′ (upstream) and 5′-TCCACGACATACTCAGCAC-3′ (downstream). PCR amplification was performed at 50°C for 5 min, 95°C for 5 min and 30 sec, then followd by 35 cycles at 95°C for 50 sec, 58°C for 50 second, and at 72°C for 50 second, followed by a melt curve from 65°C to 95°C, in increments of 0.5°C, holding for 10 sec. The relative differences in expression between groups were expressed using cycle time (Ct) values as follows: the Ct values of NR2B gene were first normalized with GAPDH of the same sample, and then the relative differences between sham and treatment groups were calculated and expressed as relative increases, setting sham group as 100%.

### Western blot analysis

Tissue samples from hippocampus were homogenized on ice in lysis buffer. The homogenate was centrifuged at 15,000 rpm for 5 min at 4°C and supernatant was removed. Protein concentrations were determined by the BCA Protein Assay Kit. Samples (30 µg) were separated by SDS-PAGE (8%) and subsequently transferred to polyvinylidene difluoride membranes (Millipore Corporation,MA, USA). The blots were blocked with 5% nonfat milk for 2 h at room temperature and incubated respectively with rabbit anti-NR2B (1∶1000; Abcam, CA, USA) and anti-β-actin (1∶2000; Cell Signaling Tech, MA, USA) primary antibody overnight at 4°C. Next day, the membranes were washed 3 times with TBS-T buffer for 15 min and incubated with the secondary goat anti-rabbit antibody conjugated with horseradish peroxidase (1∶5000; Cell Signaling Tech, MA, USA) for 2 h at room temperature. Then the immune complexes were washed 3 times with TBS-T buffer and detected using the ECL system (Millipore Corporation, MA, USA). β-actin was used as a loading control for total protein. The images of Western blot products were collected and analyzed by Quantity One V4.40 (Bio-Rad, USA).

### Immunohistochemical staining

Immunohistochemistry was used to detect the expression of NR2B in the rat hippocampus. Animals anesthetized with pentobarbital sodium (50 mg/kg, ip) were perfused through the ascending aorta with 0.9% NaCl followed by freshly prepared 4% paraformaldehyde in 0.1 M phosphate-buffered saline (PBS, pH 7.4). The rat hippocampu was carefully dissected out and were removed into the same fixative overnight (4°C) for postfixation, and then embedded in paraffin. Paraffin imbedding section was cut at 4 µm. Following deparaffinization, the 4 µm-thick paraffin sections were boiled in 0.1 mol/L sodium citrate buffer (pH 6.0) for 20 min, using primary antibodies against NR2B (1∶500, Abbiotec, American) for 30 min at 37°C. Sections were incubated in the appropriate biotinylated secondary antibodies for 30 min. Immunohistochemical controls consisting of omission of the primary antibody were performed in parallel. Sections were visualized with DAB for light microscopy examination (magnification 250× for the objective lens).

### Statistical analysis

All data are expressed as mean ± SD (standard deviation). Animals were assigned to different treatment groups in a randomized way. Repeated measures analysis of variance (ANOVA) followed by LSD test was used to examine statistical comparisons between groups. All analyses were performed using SPSS13.0. P<0.05 was considered as significant.

## Results

### Effect of senegenin on cognitive deficits induced by hepatic ischemia-reperfusion in rats

**Figure 1:**
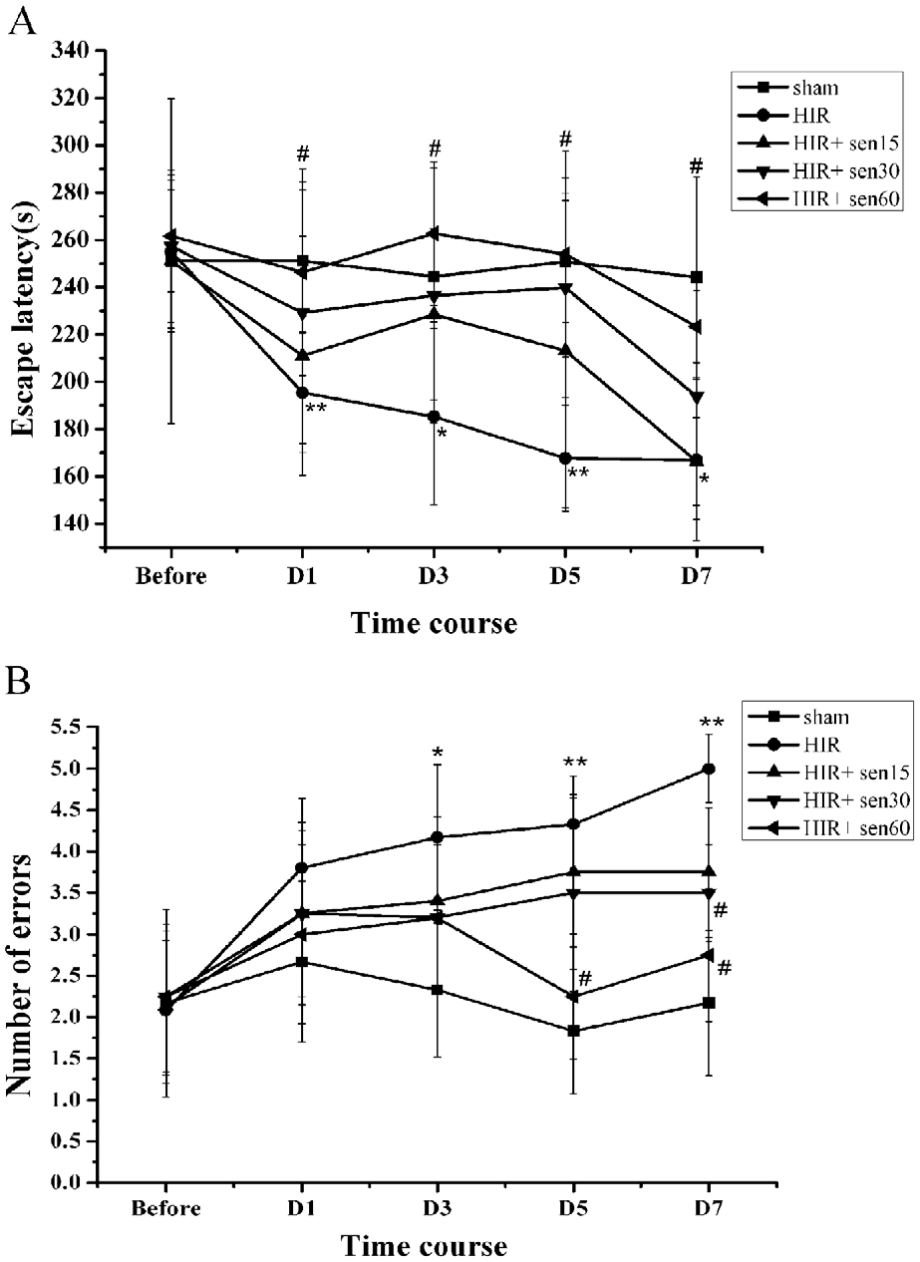
Effect of senegenin on cognitive deficits induced by hepatic ischemia-reperfusion in rats. A.Mean escape latency in the step-down test of every group at 1 (D1), 3 (D3), 5 (D5), or 7 (D7) day afte operation.B. Number of errors in Y maze test. Results are represented as mean±SD (n = 6). * P<0.05, ** P<0.01 vs sham group at the same time point; # P<0.05 vs HIR group at the same time point.

In the step-down test, the escape latency between placement on the platform and step-down onto the copper bar of the rats in the HIR group was significantly lower than that of sham group at each time point postoperation (P<0.01 at day 1,5 and P<0.05 at day 3,7 postoperation, Fig. 1A). Meanwhile, the Y maze test showed augmented number of errors of the rats in the HIR group (P<0.05 at day 3 and P<0.01 at day 5,7 postoperation, Fig. 1B).These results suggest that the ability of memory retention was impaired after HIR.

The HIR+sen15 group showed no difference with HIR group at each time point (p>0.05, Fig. 1A and B). In the HIR+sen30 group, number of errors at day 7 postoperation was significantly lower than that of HIR group (3.50±0.58 vs 5.00±0.41, P<0.05, Fig. 1B). In the HIR+sen60 group, rats exhibited an increase in escape latency (P<0.05) at each time point, as well as an decrease in number of errors (P<0.05) at day 5 (2.25±0.76 vs 4.33±0.58) and 7 (2.75±0.80 vs 5.00±0.41) postoperation.

### Effect of senegenin on the expression of NR2B mRNA in the rat hippocampus

**Figure 2:**
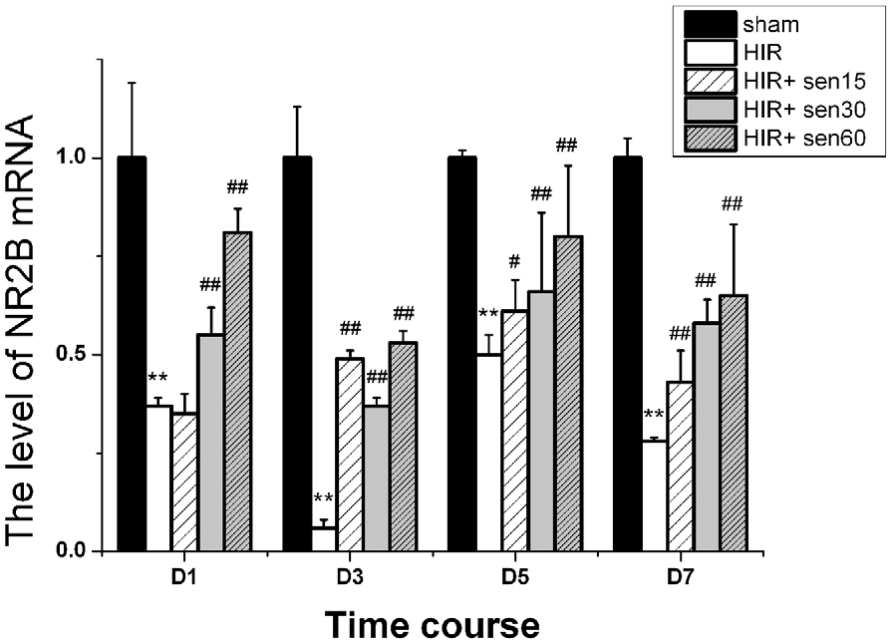
NR2B mRNA relative expression in rats hippocampus. RT-PCR images for NR2B mRNA extracted from the hippocampus of every group at 1 (D1), 3 (D3), 5 (D5), or 7 (D7) day postoperation. NR2B mRNA values were first normalized with GAPDH of the same sample, and then expressed as mean relative values compared with the sham group (set to 1). ** P<0.01 vs sham group at the same time point; # P<0.05, ## P<0.01 vs HIR group at the same time point.

Because NR2B has been previously demonstrated to play a critical role in synaptic plasticity, learning and memory, we next test changes in the expression of NR2B mRNA in rat hippocampus by RT-PCR. In the HIR group, an decreased mRNA expression of NR2B at each time point postoperation was observed (P<0.01 vs the sham group, Fig. 2), and at day 3 postoperation decreased to the lowest (0.06±0.02). The HIR+15 group showed no differences with HIR group at day 1 postoperation (0.35±0.05 vs 0.37±0.02, P>0.05, Fig. 2), but then NR2B expression was gradually increased over time until day 7. Meanwhile, after treatment with senegenin at the dose of 60 mg/kg, the maximum up-regulation of NR2B mRNA was observed at each time point.

### Effect of senegenin on the expression of NR2B protein in the rats hippocampus

**Figure 3:**
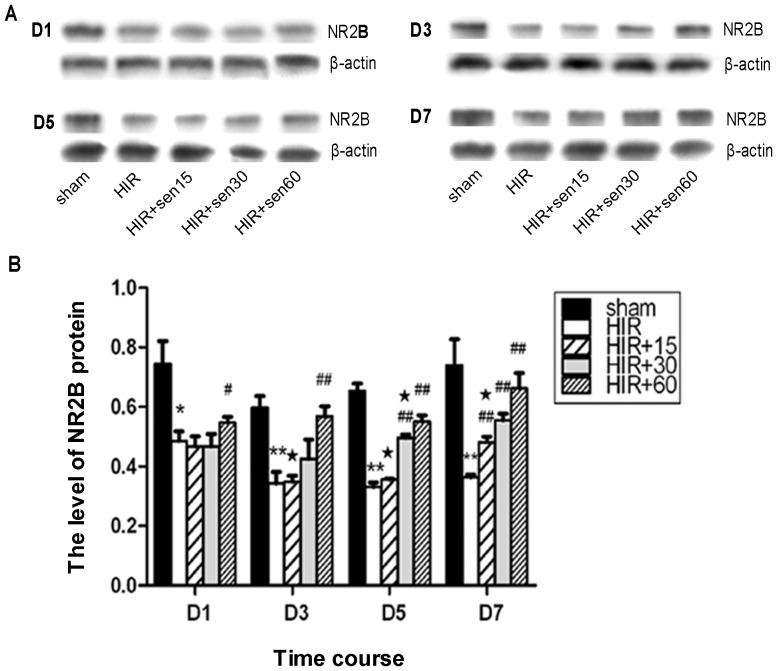
Expression of NR2B protein in rats hippocampus. A,Werstern blot images of NR2B protein extracted from the hippocampus of every group at 1 (D1), 3 (D3), 5 (D5), or 7 (D7) day afte operation. B, Statistical analysis of the relative optical density normalized to housekeeping gene β-actin (mean±SD,N = 3). * P<0.05, ** P<0.01 vs sham group at the same time point; # P<0.05, ## P<0.01 vs HIR group at the same time point; ★ P<0.01 vs HIR+60sen group at the same time point.

To explore the mechanism of senegenin on the attenuation of cognitive dysfunction induced by hepatic ischemia-reperfusion, the expressions of NR2B was detected by Western blot. Consistent with the mRNA expressions, HIR dramatically reduced the expression of NR2B at day 1 postoperation (0.49±0.03 vs 0.75±0.08, P<0.05, Fig. 3B) in rat hippocampus. At day 1 and 3 postoperation, there was no significant difference in NR2B expression between HIR+sen15 or 30 group and HIR group (P>0.05, Fig. 3B). Treatment with 15 mg/kg senegenin began to take effect at day 7 postoperation, while 30 mg/kg at day 5 and 60 mg/kg at day 1 postoperation. Moreover, at day 5 postoperation, the up-regulation of NR2B expression in HIR+sen60 group (0.55±0.02) was significantly higher (P<0.05, Fig. 3B) than that of HIR+15 (0.36±0.00) or HIR+sen30 group (0.50±0.01) respectively.

### Effect of senegenin on the expression of NR2B protein in the CA1 area of hippocampus in rats

**Figure 4:**
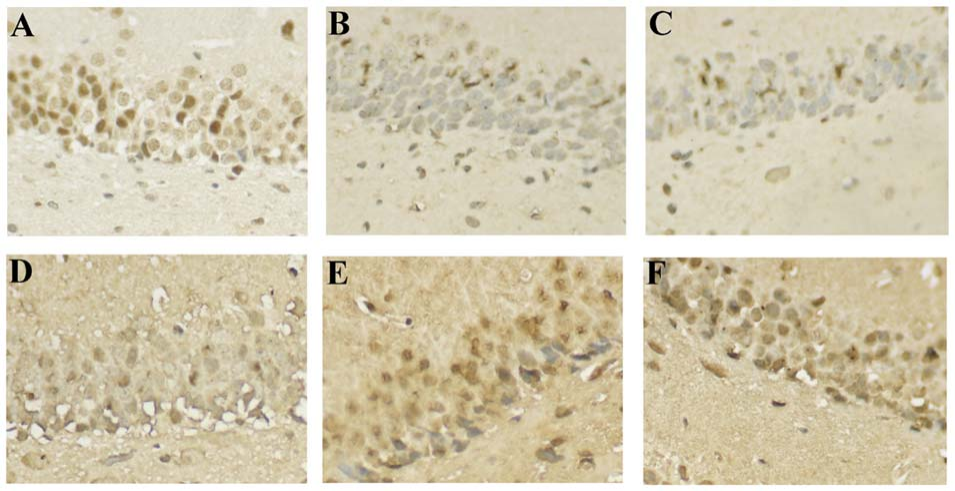
NR2B expression in the CA1 area of hippocampus in rats. Representative photomicrographs of the CA1 area of hippocampus illustrating expression of NR2B from sham group (A) at day 1 postoperation, or HIR group tested at day 1 (B), or 7 (C) postoperation or from HIR+15 (D), HIR+30 (E), or HIR+60 (F) groups tested at day 7 postoperation.

Immunohistochemical staining for NR2B is illustrated in the CA1 area of hippocampus in rats. NR2B immunoreactivity was reduced in the HIR rats (Fig. 4B and C) when compared with rats from sham group (Fig. 4A). Upregulation of NR2B expression was seen after treatment with senegenin at the dose of 15 mg/kg at day 7 postoperation (Fig. 4D). Moreover, NR2B expression was significantly increased in HIR+30 (Fig. 4E), and HIR+60 (Fig. 4F) group at day 7 postoperation.

## Discussion

In the present study, we found that hepatic ischemia-reperfusion induced cognitive impairment in rats, as well as a decrease of NR2B expression in the hippocampus. Senegenin had protective effects against memory impairment in a dose and time dependent manner. Meanwhile, it can increase the expression of NR2B in the hippocampus.

We used a model of hepatic ischemia-reperfusion (a common type of procedure during liver surgery) in rats to carry out our studies of postoperative cognitive dysfunction. The result showed that the ability of memory retention in the step-down and Y maze test was impaired after HIR, revealing hippocampal-dependent memory impairment. Several studies in vivo have clearly demonstrated inflammation plays a pivotal role in the pathogenesis of POCD [15], [16], [17], [22]. Following peripheral surgery, hippocampus shows inflammatory changes, demonstrated by a local increase in the transcription and expression of IL-1β as well as reactive microgliosis [22]. Functional inhibition of IL-1β, both in mice pretreated with IL-1 receptor antagonist and in IL-1R−/− mice, mitigated the neuroinflammatory effects of surgery and memory dysfunction [23]. The immune system and the central nervous system form a bidirectional communication network. Systemic cytokines, in particular TNFa, augment blood–brain barrier (BBB) permeability, allowing peripheral immunocompetent cells to invade the central nervous system (CNS), then affecting hippocampal networks, synapses and ultimately affecting memory function [24]. Treatment of mice with prophylactic administration of cholinergic agonists or a monoclonal antibody to TNF-α prevented neuroinflammation in the hippocampus and cognitive decline following surgery [17], [24]. Besides immune system, the brain receives sensory information related to the metabolic and other functions of the liver via neuronal pathways including both vagal and spinal visceral afferents [25], [26]. The aim of the present research was to understand the mechanisms whereby the neuron is impaired by the inflammatory response and how this can be resolved. Our results showed that the expression of NR2B is reduced in rat hippocampus after hepatic ischemia-reperfusion. Consistent with the behavioral results, a significant decrease in NR2B mRNA and protein expression in rat hippocampus was observed after HIR, and immunohistochemical studies showed a decrease in hippocampus pyramidal cells of CA1. These findings indicated that dysfunctional glutamate neurotransmission is involved in POCD, and NR2B is an important target for the prevention and treatment of POCD.

In our study, treatment with senegenin at a dose of 60 mg/kg significantly mitigated the cognitive dysfunction and attenuated the reduction of NR2B expression induced by HIR. Rats were administered of senegenin by gavage once per day started one day before surgery and thereafter continuously until sacrifice. The HIR+sen15 group showed no statistical difference compared with HIR group at each time point in behavioral memory, and the HIR+sen30 group showed protective effect just in Y maze test at day 7 but had no statistical difference in the step down test at each time point. Moreover, the maximum up-regulation of NR2B expression was observed in the HIR+sen60 group when compared with the other treatment groups. These data implicates that senegenin is an active compound from Polygala tenuifolia root and plays a neuroprotective role in HIR via increasing NR2B expression in a dose and time dependent manner. Our findings are in agreement with previous genetic studies that forebrain NR2B overexpression enhanced spatial and fear memory, and LTP induced by tetanic stimulation or repetitive stimulation were significantly enhanced as compared with wild-type littermates [27].

The N-methyl-D-aspartate (NMDA) receptor, an ionotropic glutamate receptor that is a voltage- and ligand-gated one and shows high permeability to *Ca^2+^* [28], [29], is composed of an principal subunit NR1 and at least of one of the modulatory subunits including NR2 (with A, B, C and D subtypes) and NR3 (with A and B subtypes) [30]. The NR2A and NR2B subunits are predominant subunits in excitatory pyramidal cells of the cortex and hippocampus to form the receptor complex with the NR1 subunit and to underlie the receptor's coincidence detection function [31]. A previous study showed that systemic injection of NR2B receptor antagonist ifenprodil before training led to a dose-dependent impairment in the acquisition of auditory and contextual fear conditioning [32]. What's more, augmentation of NR2B receptor membrane transportation [33], or reduction of its degradation [34] leads to enhancement of synaptic plasticity and memory. Conversely, a deficit in NR2B expression may play a critically important role in age-related cognitive decline. Using antisense oligonucleotides to specifically knock down NR2B expression in the hippocampus of young rats abolished NMDA-dependent long-term potentiation (LTP), and impaired spatial learning, just as aged rats known to have deficits in spatial learning behavior and in NR2B protein expression [35]. These findings indicated that genetic up-regulation of NR2B expression in the adult cortex and hippocampus is an effective means for rejuvenating synaptic plasticity and improving the ability of memory [36].

Several studies have reported the neuroprotective and neuroregenerative effects of Polygala tenuifolia root extracts. A previous study has shown that oral administration of Polygala tenuifolia extract has therapeutic effects on memory and behavioural disorders in rats [37]. Other researchers have reported that Polygala tenuifolia root extracts is capable of reversing scopolamine induced cognitive impairment by inhibiting acetylcholinesterase activity or enhancing the cholinergic system [3]. The mechanisms of neuroprotective effects were also reported to involve potential antipsychotic [38], antistress [39], anti-inflammatory [40], antioxidative [12], and neuro-regenerative [41], which all might explain at least in part the beneficial effects of Polygala tenuifolia root extracts against neuropathologic disorders. In this study, a purified component of Polygala tenuifolia, senegenin or tenuigenin, was examined and revealed to be an effective compound in vivo. We found that treatment with senegenin at a dose of 60 mg/kg significantly attenuated the cognitive dysfunction and the maximum up-regulation of NR2B mRNA and protein expression was observed in the HIR+60 group when compared with the other treatment groups. Our novel findings suggest that senegenin could improve cognitive impairment through elevating NR2B and rise the possibility that this extract may have some protective effects against neural impairments in POCD, or other neurodegenerative diseases related to NMDA-dependent signaling pathway dysfunction, such as Alzheimer's disease, vascular dementia and age-associated cognitive decline. Furthermore, while we have found no apparent side effects of senegenin, long-term in vivo studies will be necessary to evaluate the safety of this compound.

Taken together, the present study demonstrated that dysfunctional glutamate neurotransmission is implicated in POCD, and NMDA receptor is an important target for the prevention and treatment of POCD. Senegenin played a neuroprotective role in HIR via increasing NR2B expression in a dose and time dependent manner. Future studies may examine the underlying mechanism that are involved in the down regulation of NR2B in the development of POCD, which may provide a new strategy for treatment of POCD.
